# Biochemical and clinical response after umbilical cord blood transplant in a boy with early childhood‐onset beta‐mannosidosis

**DOI:** 10.1002/mgg3.712

**Published:** 2019-05-21

**Authors:** Troy C. Lund, Weston P. Miller, Julie B. Eisengart, Katrina Simmons, Laura Pollard, Deborah L. Renaud, David A. Wenger, Marc C. Patterson, Paul J Orchard

**Affiliations:** ^1^ Division of Pediatric Blood and Marrow Transplant University of Minnesota Minneapolis Minnesota; ^2^ Sangamo Therapeutics Richmond California; ^3^ Division of Clinical Behavioral Neuroscience, Department of Pediatrics University of Minnesota Minneapolis Minnesota; ^4^ Sanofi, Rare Disease Division Sanofi Genzyme US Bridgewater New Jersey; ^5^ Biochemical Genetics Laboratory Greenwood Genetic Center Greenwood South Carolina; ^6^ Department of Neurology, Department of Clinical Genomics, Department of Pediatrics Mayo Clinic Rochester Minnesota; ^7^ Lysosomal Diseases Testing Laboratory, Department of Neurology Sidney Kimmel Medical College, Thomas Jefferson University Philadelphia Pennsylvania; ^8^ Division of Child and Adolescent Neurology Mayo Clinic Rochester Minnesota

**Keywords:** beta‐mannosidase, beta‐mannosidosis, storage disease, umbilical cord blood transplant

## Abstract

**Background:**

Deficiency in the enzyme β‐mannosidase was described over three decades ago. Although rare in occurrence, the presentation of childhood‐onset β‐mannosidase deficiency consists of hypotonia in the newborn period followed by global development delay, behavior problems, and intellectual disability. No effective pharmacologic treatments have been available.

**Methods:**

We report 2‐year outcomes following the first umbilical cord blood transplant in a 4‐year‐old boy with early childhood‐onset disease.

**Results:**

We show restoration of leukocyte β‐mannosidase activity which remained normal at 2 years posttransplant, and a simultaneous increase in plasma β‐mannosidase activity and dramatic decrease in urine‐free oligosaccharides were also observed. MRI of the brain remained stable. Neurocognitive evaluation revealed test point gains, although the magnitude of improvement was less than expected for age, causing lower IQ scores that represent a wider developmental gap between the patient and unaffected peers.

**Conclusion:**

Our findings suggest that hematopoietic cell transplant can correct the biochemical defect in β‐mannosidosis, although preservation of the neurocognitive trajectory may be a challenge.

## 1 INTRODUCTION

β‐Mannosidosis is a rare, autosomal‐recessive disorder of glycoprotein catabolism resulting from insufficient lysosomal β‐mannosidase activity (OMIM#248510) (Cooper, Hatton, Thornley, & Sardharwalla, 1990; Michalski & Klein, 1999). First described in a human case just over three decades ago(Wenger, Sujansky, Fennessey, & Thompson, 1986), relatively few reports of human disease have emerged since that time, with phenotypic variability in age‐of‐onset, rate of progression, and clinical severity (Gort et al., 2006; Labauge et al., 2009; Riise Stensland et al., 2008; Sedel et al., 2006). Within kinships, disease may not uniformly manifest, suggesting a role for modifiers beyond residual enzyme activity (Alkhayat et al., 1998; Dorland et al., 1988; Kleijer et al., 1990). In severe cases, early childhood‐onset disease can lead to hypotonia, dysostosis, intellectual disability, behavioral abnormalities, and peripheral neuropathy (Bedilu et al., 2002; Kleijer et al., 1990; Poenaru, Akli, Rocchiccioli, Eydoux, & Zamet, 1992; Wijburg et al., 1992). Patients are primarily diagnosed by measuring β‐mannosidase activity in plasma or leukocytes and present with increase urinary disaccharides particularly mannosyl‐N‐acetylglucosamine. No definitive therapies exist beyond symptomatic treatment.

Allogeneic hematopoietic cell transplantation (HCT) benefits various neuropathic lysosomal and peroxisomal inherited metabolic disorders (IMD) on the basis of metabolic cross‐correction. In the modern pediatric IMD transplant era, the use of human leukocyte antigen (HLA) histocompatible umbilical cord blood (UCB) is associated with favorable rates of engrafted survival (Boelens et al., 2013; Martin et al., 2013; Miller et al., 2011). A recent report documents particularly favorable outcomes with HLA‐matched umbilical cord blood transplant (UCBT) in IMD (Mallhi et al., 2017). Similarly, low transplant regimen‐related toxicities have been reported with busulfan and fludarabine conditioning (Bartelink et al., 2014). Scant reports document benefit following HCT for other glycoproteinoses, but none describe transplantation for β‐Mannosidosis (Grewal et al., 2004; Miano et al., 2001; Mynarek et al., 2012; Vellodi et al., 1995). We report 2‐year outcomes following HLA geno‐identical UCBT in a boy with early childhood‐onset disease.

## 2 CASE DESCRIPTION

**Figure 1:**
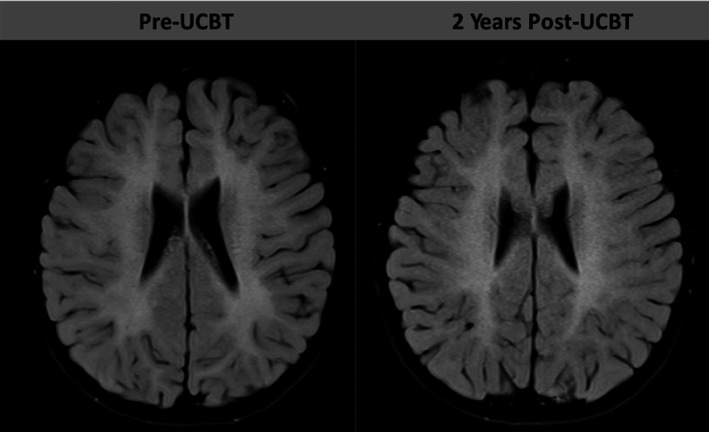
Representative axial FLAIR sequences from brain MRI. Left MRI was performed at age 4 years, 8 months (pre‐UCBT) and at age 6 years, 8 months (2 years post‐UCBT) in a boy with β‐mannosidosis

**Figure 2:**
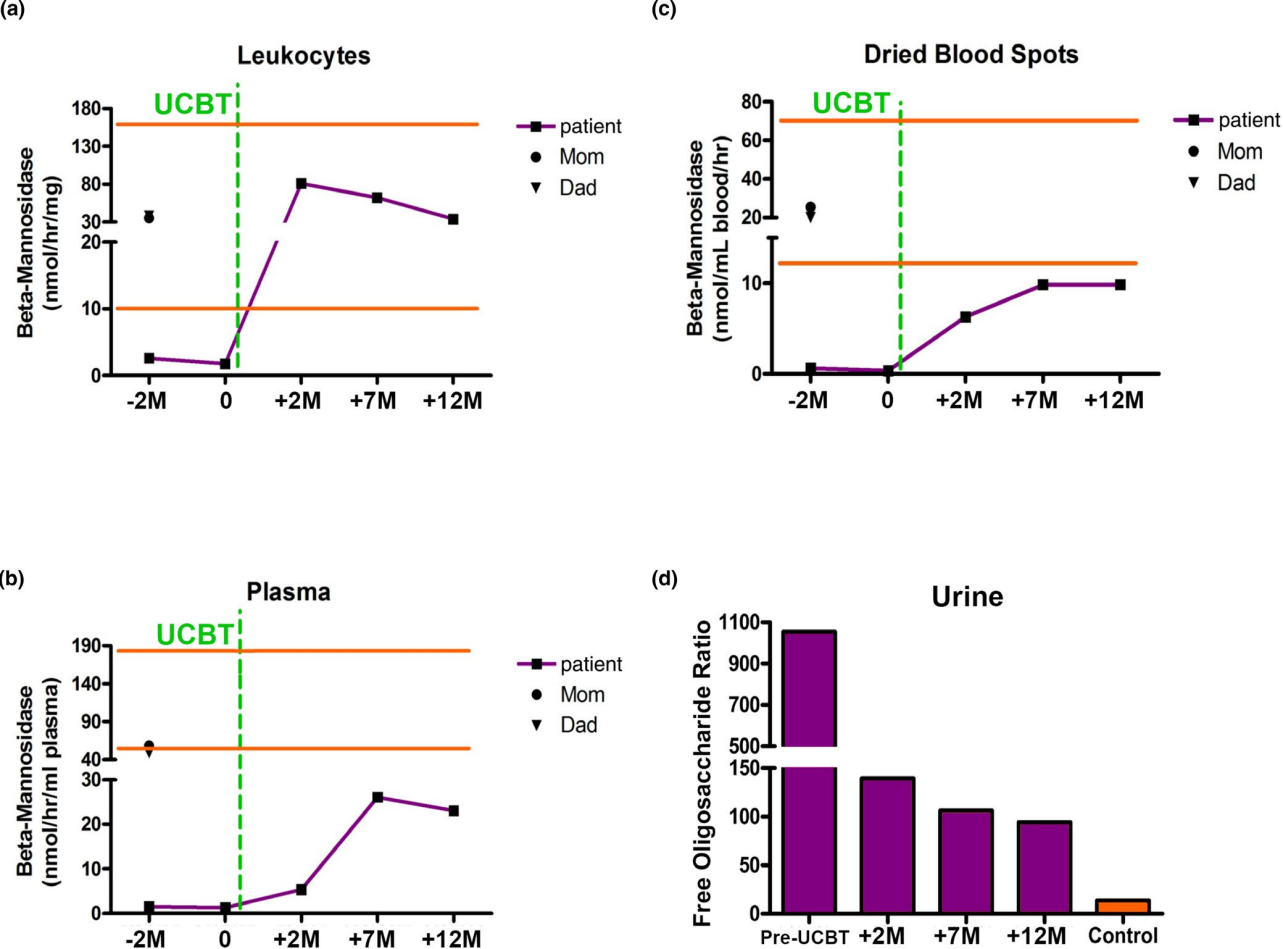
Mannosidase biomarkers pre‐ and post‐UCBT. Panels A‐C show beta‐mannosidase activity in the various biologic specimens indicated. The horizontal lines represent the limits of the normal range. Panel D shows urinary free oligosaccharide levels relative to an internal standard measured by LC‐MS/MS specific for beta‐mannosidosis. The time scales are given in months relative to UCBT

A 4‐year, 6‐month‐old male was referred to us with newly diagnosed β‐mannosidosis. His birth history was notable for maternal preeclampsia necessitating C‐section at term. After an uneventful infancy, he was found to have hypotonia at 8 months of age. He rolled at 11 months, sat alone at 12 months and walked at 22 months. He first spoke at 30 months. At age 1 year, macrocephaly was noted, and an MRI revealed mild‐to‐moderate cerebral hypomyelination. He was diagnosed with Autism Spectrum Disorder at age 2. Due to persistent language and motor developmental delays in the face of multiple rehabilitative therapies, repeat MRI at age 4 years, 3 months re‐demonstrated poor myelination with symmetric T2 hyperintensities in the deep and subcortical white matter as well as the posterior limbs of the internal capsule (Figure 1). Additionally, he was noted to have a head circumference at the 93rd percentile. Given the constellation of findings, the possibility of a metabolic storage disease was part of the differential diagnosis, and a leukocyte oligosaccharidosis screen was performed that showed low leukocyte β‐mannosidase activity at 2.62 nmol hr^‐1^ mg^‐1^ (normal range 10–162.4 nmol hr^‐1^ mg^‐1^, Figure 2). Sequencing of MANBA (OMIM #248510) revealed compound heterozygous mutations: NP_005899.3:p.(Trp192Ter) (known pathogenic) and NP_005899.3:p.(Arg500His)(novel, but predicted to be pathogenic).

His exam prior to UCBT showed dysarthria, mild hypotonia, mild dysmetria, and wide‐based gait with external hip rotation. An EEG, nerve conduction study (NCS), tympanogram, and otoacoustic emissions evaluation were all normal. Initial neurocognitive assessment prior to UCBT indicated slightly below average intellectual functioning and impaired language functioning.

## 3 METHODS

Transplant proceeded using a University of Minnesota Institutional Review Board approved protocol following parental consent. The conditioning regimen consisted of thymoglobulin (2.5 mg kg^‐1^dose^‐1^) IV once daily (−8, −7, −6 and −5); and busulfan and fludarabine (40 mg m^‐2^ dose^‐1^), each IV once daily (−5, −4, −3 and −2). Daily busulfan pharmacokinetics were monitored for a total exposure of 90 mg h L^‐1^. The graft was an 8/8 allele‐matched (HLA‐A, ‐B, ‐C and –DRB1) unrelated umbilical cord blood unit with a total nucleated cell count (TNC) of 14 × 10^7^ cells/kg. Graft versus host disease (GvHD) prophylaxis was with cyclosporine (days −2 to +180; goal trough 200–400 µg/L) and prednisolone (2 mg/kg through day + 28, then tapered to off over 2 weeks). Antimicrobial prophylaxis was with fluconazole and co‐trimoxazole. Engraftment kinetics, immune reconstitution, and donor hematopoietic chimerism were longitudinally monitored.

Leukocyte, plasma, and dried blood spot β‐mannosidase activity were measured before and after transplantation using the 4‐methylumbelliferyl substrate as previously described (Wenger et al., 1986). Urinary mannosyl‐N‐acetylglucosamine was measured using liquid chromatography tandem mass spectrometry (Huang, Cathey, Pollard, & Wood, 2018). Baseline and posttransplantation neuropsychological assessments of intellectual function, adaptive behavior (i.e., functional life skills), memory, and visual‐motor skills (i.e., drawing; Beery & Beery, 2010; Driessen et al., 2003; Kan, Melamed, & Offen, 2007; Kopen, Prockop, & Phinney, 1999), as well as brain MRI, were part of standard protocol for HCT. The neuropsychological evaluations use an age‐based standardized score with a population mean of 100 (standard deviation = 15), and the descriptive ranges are: average (85–115), below average (70–84) and impaired (<70).

## 4 RESULTS AND DISCUSSION

Neutrophil and platelet engraftment occurred at days +13 and +21, respectively. Full (100%) donor myeloid chimerism was observed on day +21 and continues at most‐recent follow‐up (2 years). Donor T‐cell chimerism was initially 38% at day +21 and is 91% at 2 years post‐UCBT. At 1‐year posttransplantation, absolute T‐ and B‐cell absolute counts returned to the normal range (which were similar to pretransplantation levels). No GvHD was observed and no infectious complications developed. Leukocyte β‐mannosidase activity normalized at first posttransplant assessment (81.1 nmol hr^‐1^ mg^‐1^) and remains normal at 2 years post‐UCBT, and a simultaneous increase in plasma β‐mannosidase activity and dramatic decrease in urine free oligosaccharides were also observed (Figure 2).

Within the first several months of transplantation, the child experienced migraine headaches, nystagmus, worsening ataxia, loss of independent feeding, increased fearfulness and anxiety, anorexia requiring parenteral nutrition, and intentional tremor (responsive to propranolol). Brain imaging again showed T2 white matter abnormalities that were stable in size and extent. By 6 months post‐UCBT, symptoms stabilized, and the child was off parenteral nutrition support. One year after UCBT, significant improvements in gait, headache, tremor, nystagmus, fearfulness, and anxiety were noted. Brain MRI again remained stable and NCS remained normal (not shown). Two years after UCBT, continued improvements were noted with respect to anxiety and gait. There were residual nystagmus, intention tremor, mild leg spasticity, and imbalance symptoms. Brain MRI re‐demonstrated stabilization of white matter disease indicated by no change in the T2 signal abnormalities seen previously (Figure 1).

Neuropsychological assessment (Table 1) revealed pre‐UCBT adaptive behavior functioning was average overall, although there was below average adaptive motor functioning. At 6 months following UCBT, adaptive behavior was significantly impaired, yet it returned to baseline 2 years post‐UCBT with persistent impairment in motor functioning (Table 1). In terms of intellectual function, although overall IQ score declined from baseline to 2 years post‐UCBT, analysis of raw score test points earned at each administration revealed either similar or greater points achieved at follow‐up. However, the magnitude of improvement was less than expected for age, causing the lower IQ score which represents a wider developmental gap between the patient and unaffected peers. This phenomenon is also true for a decline in visual‐motor integration score. Specifically, this child was initially mildly below average on this test, then impaired at 2‐year follow‐up, but he actually earned more raw score test points at 2‐year follow‐up than baseline. In fact, ataxia at 6 months post‐UCBT made completion of this test impossible, and thus the achievement of greater points on this test than baseline at 2 years post‐UCBT is notable. In contrast, memory clearly declined from the average range at both baseline and 6 months follow‐up, to the impaired range at 2 years follow‐up, with less than half the raw score test points earned at baseline. Memory challenges can be one of many factors contributing to slowed neurocognitive development.

**Table 1:** Neuropsychological profile at baseline and longitudinally following UCBT in a 4‐year‐old boy with β‐Mannosidosis

Parameter	Pre‐UCBT (age = 4 years, 4 m)	6 months Post‐UCBT (age = 5 years, 2 m)	2 years Post‐UCBT (age = 6 years, 8 m)
Adaptive behavior skills			
Communication	100	79	102
Daily living skills	95	69	89
Socialization	95	85	103
Motor	70	37	61
Adaptive behavior composite	88	64	86
Cognition			
Nonverbal IQ	84 (41)	N/A	64 (43)
Verbal IQ	78 (37)	N/A	75 (57)
Full Scale IQ	80	N/A	68
Memory	100 (47)	100 (51)	65 (21)
Visual‐motor skills	80 (7)	Unable	64 (10)

Note

Scores are age normalized (100 ± 15, with 85–115 the normal range). Scores in parentheses represent the number of items correctly answered on the test domains, that is, raw score. The Full Scale IQ score is a transformed score that is not directly derived from raw score points, and thus no parenthetical number is applicable.

Early childhood‐onset β‐mannosidosis, though extremely rare, can manifest significant developmental delay and neurologic abnormalities. Today no disease‐specific therapies exist. Allogeneic HCT has documented efficacy in numerous lysosomal enzymopathies, as donor‐derived hematopoietic cells are able to partially restore substrate catabolism. Further, HCT for lysosomal disorders involving neurocognitive deterioration has been shown to arrest decline, although it has not reversed neurocognitive loss (Aldenhoven et al., 2015; Hobbs et al., 1981; Krivit, Aubourg, Shapiro, & Peters, 1999; Shapiro et al., 2015). While no reports of HCT for β‐mannosidosis exist in the literature, several reports catalog benefit in other disorders of glycoprotein metabolism, suggesting potential benefit for the disease. As favorable engrafted survival rates are observed with HLA geno‐identical UCBT in the modern era, we deemed the risks of treatment failure to be acceptably low.

Despite rapid normalization of leukocyte β‐mannosidase activity, our patient experienced acute clinical neurological worsening in the immediate posttransplantation period, before gradually returning to near‐baseline performance. Such posttransplantation course (acute, transient neurologic worsening) is not unusual for patients with other neuropathic IMD and may represent reversible effects from medications for conditioning or use of calcineurin inhibitors in the prevention of GvHD.

Consistent with stable MRI between baseline and 2‐year follow‐up, the boy's neurocognitive performance does not provide evidence of active deterioration or skill loss, with the exception of memory function. Overall, his neurocognitive trajectory has not aligned with age‐level expectations, and therefore his scores are all generally lower. This pattern of lower post‐UCBT performance even after an intact pre‐UCBT baseline is seen in other neuropathic IMD such as Hurler syndrome and cerebral adrenoleukodystrophy (Aldenhoven et al., 2015; Coletti et al., 2015; Grosse, Lam, Wiggins, & Kemper, 2017; Pierpont et al., 2017; Shapiro et al., 2015). Longer term examination of neurocognitive function in other IMD post‐HCT suggests stabilization of neurocognition, provided that intervention occurred early enough in the disease process. Given the progressive nature of these conditions, earlier treatment is associated with better neurocognitive outcomes, as disease burden is interrupted prior to a severe level of accumulation and neurocognitive damage(Aldenhoven et al., 2015; Grosse et al., 2017; Muenzer, Wraith, Clarke, & International Consensus Panel on Management and Treatment of Mucopolysaccharidosis I, 2009; Pierpont et al., 2017; Poe, Chagnon, & Escolar, 2014; Shapiro et al., 2015). As this is the first report of UCBT for β‐mannosidosis, it will be critical to pursue further follow‐up in this child to determine if he continues to acquire neurocognitive skills, versus a developmental stagnation or actual skill loss. This follow‐up may help to clarify the impact of his baseline age and disease severity on his longer term outcomes. It may also clarify whether relative gains in other aspects of his overall neurologic status might be possible with sustained provision of functional enzyme from the UCB graft.

## CONFLICT OF INTEREST

The authors have no conflict of interest to declare.
